# New understanding of gut microbiota and colorectal anastomosis leak: A collaborative review of the current concepts

**DOI:** 10.3389/fcimb.2022.1022603

**Published:** 2022-11-01

**Authors:** Yang Liu, Bowen Li, Yunwei Wei

**Affiliations:** ^1^ Pancreatic and Gastrointestinal Surgery Division, HwaMei Hospital, University of Chinese Academy of Science, Ningbo, China; ^2^ Ningbo Clinical Research Center for Digestive System Tumors, Ningbo, China; ^3^ Department of Oncology and Laparoscopy Surgery, The First Affiliated Hospital of Harbin Medical University, Harbin, China

**Keywords:** anastomosis leak, gut microbiota, pathogen colonization, extracellular matrix, adhesin, collagen degradation

## Abstract

Anastomotic leak (AL) is a life-threatening postoperative complication following colorectal surgery, which has not decreased over time. Until now, no specific risk factors or surgical technique could be targeted to improve anastomotic healing. In the past decade, gut microbiota dysbiosis has been recognized to contribute to AL, but the exact effects are still vague. In this context, interpretation of the mechanisms underlying how the gut microbiota contributes to AL is significant for improving patients’ outcomes. This review concentrates on novel findings to explain how the gut microbiota of patients with AL are altered, how the AL-specific pathogen colonizes and is enriched on the anastomosis site, and how these pathogens conduct their tissue breakdown effects. We build up a framework between the gut microbiota and AL on three levels. Firstly, factors that shape the gut microbiota profiles in patients who developed AL after colorectal surgery include preoperative intervention and surgical factors. Secondly, AL-specific pathogenic or collagenase bacteria adhere to the intestinal mucosa and defend against host clearance, including the interaction between bacterial adhesion and host extracellular matrix (ECM), the biofilm formation, and the weakened host commercial bacterial resistance. Thirdly, we interpret the potential mechanisms of pathogen-induced poor anastomotic healing.

## Introduction

Anastomotic leak (AL) is a fatal postoperative complication following colorectal surgery. The incidence of AL has not substantially decreased over the last 50 years, despite advances in anastomotic techniques and perioperative care (Vallance et al., 2017). A general question for surgeons is whether there is room to prevent AL by improving surgical techniques. In patients without any known risk factors, AL still occurs. In these cases, surgeons have claimed that inadequate surgical operation could lead to poor blood supply or increased tension, which should be blamed (Shogan et al., 2013). Up to now, investigations on finding the optimal technique to rebuild the continuity on the anastomosis site to ensure proper healing are still ongoing (Kim et al., 2019). Although we have concluded that surficial technique is not all responsible, it is reasonable that a perfect anastomosis cannot be accomplished without an adequate surgical technique (Gershuni and Friedman, 2019). The ideal model is constituted by these factors, which are undoubtedly crucial for the success of anastomoses but are not the root cause. After decades of investigation, one thing is clear: no particular anastomosis construction technique is preferable (Neutzling et al., 2012; European Society of Coloproctology Collaborating G., 2018; Tsai and Chen, 2019). We must acknowledge the dilemma that, until now, there are no specific risk factors or surgical techniques that could be targeted to improve anastomotic healing (Shogan et al., 2013). The mechanism of anastomotic healing and the fundamental pathogenesis of leakage still need to be understood.

## Gut microbiota dysbiosis related to AL

For years, it has been established that there is a potential relationship between gut microbiota dysbiosis and AL (Shogan BD et al., 2015; van Praagh et al., 2016; Hyoju et al., 2018; Gershuni and Friedman, 2019; Foppa et al., 2020a). Due to the improvement and lower cost of sequencing technology over the past decade, there have been more opportunities to identify the gut microbiota and to recognize its interaction with the pathophysiological conditions of the human body, including colorectal cancer (CRC), inflammatory bowel disease (IBD), obesity, metabolic disorders, and even parenteral disease (Claesson et al., 2012; Rinninella et al., 2019). Up to now, the effects of microbiota on AL are still unclear, and extensive clinical evidence on the impact of the gut microbiota on postoperative anastomotic complications is still lacking (Russ and Casillas, 2016). In this review, we summarized the clinical investigation of the gut microbiota and AL (**Table 1**). Due to ethical and technique limitations, no study has discovered variations in the gut mucosal microbiota on the anastomosis site during the perioperative period.

**Table 1:** Clinical investigations on the gut microbiota and anastomotic leak (AL).

Reference	Year	No. of cases	Sample type	Time point	Method	Design of the study	Main findings
Mizuta et al. (2016)	2016	60	Stool	Before surgery and 1 week after surgery	16S rRNA sequencing	Patients undergoing colorectal resection were randomized to two groups before resection. One group received a probiotic supplement (*Bifidobacterium longum* BB536), preoperatively for 7–14 days and postoperatively for 14 days, while the other group received no intervention as a control. Postoperative infectious complications were the primary endpoint.	The proportions of fecal bacteria changed significantly in both groups. *Actinobacteria* increased in the probiotic group, Bacteroidetes and Proteobacteria increased in the control group, and *Firmicutes* decreased in both groups. Four patients in the control group, but none in the probiotic group, experienced postoperative anastomotic leakage (*p* = 0.10).
van Praagh et al. (2016)	2016	16 (AL = 8)	Anastomosis site tissue	During surgery	16S rRNA sequencing	Eight patients who developed AL requiring reintervention and eight matched controls without AL were compared.	Lachnospiraceaeis is higher, while the microbial diversity levels were lower in AL patients.
van Praagh et al. (2019)	2019	123 (AL = 29)	Anastomosis site tissue	During surgery	16S rRNA sequencing	Twenty-nine patients who developed AL were matched by sex, age, and preoperative chemotherapy and radiotherapy with 94 patients who did not.	In non-C-seal patients, AL development was related to low microbial diversity and high abundance of Bacteroidaceae and Lachnospiraceae. In C-seal patients, where the AL rates were slightly higher (25% *vs*. 17%), association with the gut microbiota composition was hardly detectable. A few opportunistic pathogenic taxa were associated with AL in C-seal patients, especially *Prevotella oralis*.
Shogan et al. (Shogan, et al., 2015)	2015	11 (AL = 1)	Distal and proximal end swabs	During surgery	16S rRNA sequencing	Patients undergoing colon surgery consented to participate in the study. When the operating surgeon removed the colon sample, the distal and proximal ends were immediately swabbed for 16S rRNA analysis and aerobic culture.	Disturbed microbial community structure and membership distribution in anastomotic tissues among the 11 patients. One patient who received reoperation for AL showed a ratio of Proteobacteria to Bacteroidetes of 3:1, indicative of a highly imbalanced microbiota.
Komen et al. (2014)	2014	243 (AL = 19)	Abdominal drain fluid	Postoperative days 1–5	RT-PCR for specific microbes	Patients enrolled in a multicenter prospective observational study underwent left-sided colorectal resection for malignant and benign tumors. In all patients, an intra-abdominal drain was placed during the operation. The quantitative results of the RT-PCR on days 2–5 were compared to those of day 1 to detect changes.	Increased *Escherichia coli* concentration was found in AL patients on days 4 and 5. For *Enterococcus faecalis*, this result was found for days 2–4, with the highest on day 3.
Palmisano et al. (2020)	2020	48 (AL = 5)	Stool samples	Before surgery and after neoadjuvant treatment	16S rRNA sequencing	Colorectal cancer patients were divided into an anastomotic leak group and an uneventful recovery group.	AL patients showed increased *Acinetobacter lwoffii* and *Hafnia alvei*, an array of bacterial species that promoted dysbiosis. Non-AL patients showed increased *Faecalibacterium prausnitzii* and *Barnesiella intestinihominis*, which have a protective function.
Mima et al. (2020)	2020	256	Fresh frozen tissues of colorectal cancer	During surgery	RT-PCR for specific microbes	This retrospective case–control study included colorectal cancer patients who underwent elective colorectal resection.	Patients with high *Bifidobacterium* levels are at high risk of anastomotic leakage.

**Figure 1:**
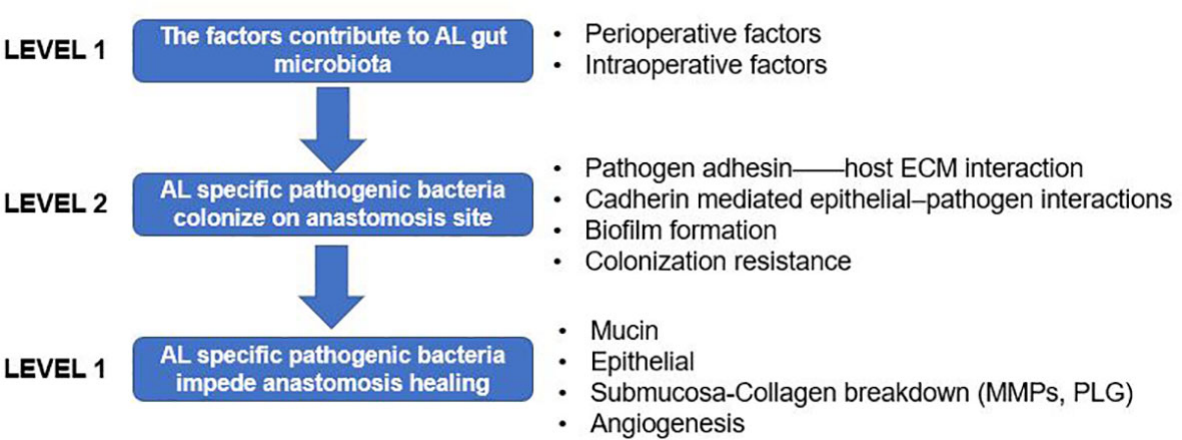
Framework of the gut microbiota and anastomotic leak (AL) presented in three levels. *Level 1*: factors that contribute to the gut microbiota; *level 2*: how pathogenic or collagenase bacteria adhere to the intestinal mucosa and avoid host clearance; and *level 3*; the mechanism by which AL-related gut bacteria interact with the host intestinal epithelial and mesenchymal cells, then affecting anastomotic healing.

It has been accepted that AL could be a product of the specific bacteria with the virulence gene (Guyton and Alverdy, 2017). A series of systemic reviews have summarized the gut microbiota and AL from different viewpoints (Bachmann et al., 2017; Gaines et al., 2018; Gershuni and Friedman, 2019; Hajjar et al., 2019; Foppa et al., 2020a). Here, we will only discuss key concepts and recent developments. We aimed to build a framework between the gut microbiota and AL in three sections (**Figure 1**): 1) factors potentially related to AL contribute to diverse gut microbiota; 2) pathogenic or collagenase bacteria adhere to the intestinal mucosa and avoid host clearance; and 3) the mechanisms by which AL-related gut bacteria affect anastomotic healing.

## Factors contributing to the varied gut microbiota potentially related to AL

Variations in the gut microbiota following surgery in CRC patients have been reviewed previously (McDermott et al., 2015; Bachmann et al., 2017). There is a theory that the microbiota can sense a dramatic environmental change from the host health status and then undergo complete compositional and functional adjustments in order to adapt (Shogan BD et al., 2014). For instance, in CRC patients undergoing surgery, the gut microbiota and metabolism showed significant changes 7 days post-surgery, characterized by decreased obligate anaerobes, enriched pathogenic bacteria, and reduced short-chain fatty acids (SCFAs) (Ohigashi et al., 2013a). A clinical study also demonstrated that the *Atopobium* cluster, *Bacillus fragilis* group, *Bifidobacterium*, and *Prevotella* decreased following CRC surgery. These obligate anaerobes are the most common bacteria and are essential in maintaining environmental stability in the human gastrointestinal tract (Hooper and Gordon, 2001). On the other hand, the facultative anaerobes, such as Enterobacteriaceae and Enterococcus, and the aerobe, such as *Pseudomonas*, which are potentially pathogenic bacteria, increased (Ohigashi et al., 2013a). Several perioperative manipulations have been recognized to induce phenotype and genotype variations in commensal microbiota, remolding them into invasive tissue-degrading pathogens during surgery (Ohigashi et al., 2013a; Shogan B et al., 2015). This phenomenon reveals that the microbial phenotype, rather than the microbial existence, is more crucial to the tissue disruption that leads to anastomotic leak (Sido et al., 2004). In this review, we focus on the preoperative and surgical factors contributing to gut microbiota alterations that are potentially related to AL.

### Preoperative factors

#### Antibiotics and MBP

In the 1970s, oral antibiotics in combination with mechanical bowel preparation (MBP) are a routine treatment to prevent infectious complications following surgery (Nichols and Condon, 1971). However, this approach remains controversial, highly debated, and poorly understood in terms of its mechanisms of action, and it was gradually discarded over time (Atkinson et al., 2015). Recent discovery has indicated that almost half of the pathogens causing serious postoperative infectious complications are antibiotic-resistant, including *Enterococcus faecalis* and *Pseudomonas aeruginosa*, which are the most common bacteria cultured from a leaking anastomosis even when strong antibiotics are used (Ohigashi et al., 2013a). The gut microbiota changes following MBP have been reviewed (Drago et al., 2019). These alterations include increased *Enterobacteriaceae* and *Proteobacteria*, but a reduction of Lactobacillus, accompanied by a decreased Gram-positive/Gram-negative ratio, similar to the profile of infectious diarrhea that can last for at least 2 weeks, and in some cases up to 4 weeks (Drago et al., 2016).

#### Neoadjuvant radiation and nonsteroidal anti-inflammatory therapy

There is still controversy regarding neoadjuvant chemoradiation therapy and AL. Radiotherapy has been demonstrated to change the gut microbiota in patients with rectal cancer, but there has been no clear demonstration of increased AL to date (Olivas et al., 2012). After neoadjuvant chemoradiotherapy, the tumor samples demonstrated significantly lower diversity and a trend toward lower unevenness. *Fusobacterium* significantly decreased following neoadjuvant chemoradiotherapy, in addition to *Peptostreptococcus*, *Parvimonas*, and *Porphyromonas*, and two genera in Lactobacillales, i.e., *Lactobacillus* and *Streptococcus*, were significantly increased (Yi et al., 2021).

A series of studies showed an increased incidence of AL due to nonsteroidal anti-inflammatory drug (NSAID) use (Peng et al., 2016; Modasi et al., 2019). However, this effect was not consistent across all NSAIDs. A review has introduced gut microbiota alterations after NSAID use in clinical and animal studies (Wang et al., 2021). The use of NSAIDs results in the proliferation of Gram-negative bacteria. Aspirin increases *Prevotella*, *Bacteroides*, Ruminococcaceae, and *Barnesiella*. Celecoxib and ibuprofen increase Acidaminococcaceae and Enterobacteriaceae. Furthermore, increases in Rikenellaceae, Propionibacteriaceae, Puniceicoccaceae, and Pseudomonadaceae were observed after ibuprofen intake. However, this gut microbiota alteration varied between men and women (Edogawa et al., 2018). It should be noted that the type of NSAID rather than the amount taken caused the greatest differences in the microbiome.

### Intraoperative factors

#### Poor perfusion and hypoxia

It is generally accepted that adequate tissue perfusion is critical for anastomotic healing. However, there is evidence that, even with minimal flow, anastomotic tissue can heal adequately (Kashiwagi, 1993). Histological analysis of anastomotic tissues excised during emergency surgery for leakage repair in 14 patients found no indication of inadequate blood flow (Schouten et al., 2014). It is reasonable to think that hypoxia is directly detrimental to low blood perfusion. In the study by Shogan et al. performed in mice, devascularization of a colon tube led to poor anastomotic healing. However, hypoxia was not associated with the anastomotic healing grade. In addition, histological examination failed to provide evidence of tissue hypoxiation (Shakhsheer et al., 2017).

Tissue ischemia could impact the local microbiota composition. In a mouse model, mesenteric ischemia followed by reperfusion increased *Escherichia coli* and decreased *Lactobacillus* in the ileum and colon, which persisted for approximately 6 h after recovery (Wang et al., 2012). This shift was accompanied by a breakdown of the intestinal barrier and loss of mucosal integrity, which permitted the translocation of potentially pathogenic bacterial species (Guyton and Alverdy, 2017). An interesting study found that human intestinal epithelial cells release soluble factors when subjected to hypoxia and reoxygenation. These factors can induce *P. aeruginosa* to express the potent barrier-dysregulating protein PA-I lectin/adhesin (Patel et al., 2007). In addition, factors such as adenosine and dynorphin can transition *P. aeruginosa* to a more aggressive and barrier-disrupting phenotype with high collagen-degrading activity by activating quorum sensing (Alverdy and Chang, 2008; Shogan B et al., 2015). Devascularized conditions support a favorable environment for *E. faecalis* to transform into phenotypes that can promote anastomotic leaks, independent of tissue hypoxia (Shogan B et al., 2015). The explanation for this might be that the low perfusion caused by devascularization is not bound to hypoxia. In addition, to a certain degree, hypoxia stimulates the cells to produce angiogenic growth factors. In contrast, only severe tissue hypoxia combines with the lactic acid produced by bacteria to lower the tissue pH, which contributes to tissue breakdown. Inadequate nutrients and immune component transportation to the anastomosis site caused by low perfusion might be another explanation for this phenomenon. Two studies demonstrated that, even in ischemic tissues, AL does not occur without intestinal bacteria (Shogan B et al., 2015). These results indicate the essential role of the gut microbiota in ischemia-related AL. The hypoxia-induced dysbiosis of the microbiota composition and virulence acquisition of specific pathogens should be taken into consideration when studying AL under low perfusion and hypoxia.

#### Inflammation

Inflammation plays a role in AL. Nevertheless, the cellular and molecular aspects of inflammation related to AL remain to be discovered. Inflammation of the gut wall is attributed to tissue injury and intestinal manipulation. In addition, patients with pre-surgery intestinal inflammation such as IBD should also be considered.

##### Preexisting intestinal inflammation

A number of patients with IBD need ileocecal resection. Steroid use and preoperative abscess in these patients are associated with higher anastomotic rates (Tzivanakis et al., 2012). Thus, the preexisting inflammatory condition is a crucial issue in identifying the effect of surgery on abnormal intestinal wound healing (Binnebösel et al., 2014).

The pathogenesis of IBD is driven by an abnormal and prolonged T-cell-mediated immune response directed toward the commensal gut microbiota that occurs in genetically susceptible individuals. In addition, patients with IBD display a reduction in SCFA-producing bacteria such as *Faecalibacterium prausnitzii*, which is well known to have anti-inflammatory properties through its ability to produce butyrate, allowing for T regulatory cell and T helper 17 regulation (Zhou et al., 2018).

Strong evidence indicates intestinal microbiota dysbiosis as responsible for triggering IBD (Lavelle and Sokol, 2020). The composition of the microbial taxa in patients with IBD has been extensively studied. The gut microbiota of these patients demonstrate low diversity, specific shifts in the proportion of taxa, and an altered functional capacity; all of these characteristics can be found in patients with AL (Kostic et al., 2014). These include increased Gammaproteobacteria, *E. faecalis*, *E. coli*, and *Fusobacterium* species and reduced *Bacteroides*, *Firmicutes*, *Clostridia*, Ruminococcaceae, *Bifidobacterium*, and *Lactobacillus* (Kostic et al., 2014; Lengfelder et al., 2019). The metalloprotease GelE, produced by commensal strains of *E. faecalis*, contributes to the development of AL by activating matrix metalloproteinase 9 (MMP-9) (Shogan BD et al., 2015). The relative abundance of the mucin-degrading *Ruminococcus* has been associated with AL, similarly to the case in IBD (van Praagh et al., 2016). *Fusobacterium nucleatum* aggravates the progression of IBD. It can induce the activation of macrophages and then promote phenotype transformation *via* the AKT2 signaling pathway. These effects can damage the intestinal mucosal barrier, which is destructive for wound healing (Liu et al., 2019).

Other changes lead to the loss of protective factors, such as SCFAs, and an increase in pro-inflammatory factors, such as lipopolysaccharide (LPS), further leading to an inflammatory *versus* tolerogenic milieu (Kostic et al., 2014; Vatanen et al., 2016). In addition, when antibiotics are used in IBD therapy when infectious complications are suspected and before surgical interventions, the already destroyed gut microbiota can become weaker, characterized by low diversity and depleted SCFA-producing taxa, which could also lead to AL indirectly (Ianiro et al., 2016). However, whether this preexisting microbiota dysbiosis contributes to AL is unknown.

##### Surgical tissue injury and wound healing-related inflammation

Surgical manipulation of the intestinal tube activates local inflammatory response within the muscular layer (Türler et al., 2002). This inflammatory overflow includes resident muscularis macrophage activation, immunocompetent leukocyte extravasation, and a cascade of cytokine fluid (Sido et al., 2004). Matrix metalloproteinase (MMP) is upregulated following intestinal manipulation (Moore et al., 2011). Excessive inflammatory mediators are supposed to contribute to AL directly or indirectly (Pantelis et al., 2011). However, gut microbiota alteration due to surgical manipulation has not been studied in depth to date.

The classic course of wound healing includes inflammation, proliferation, and remodeling stages, which have been extensively studied in the skin (Gurtner et al., 2008), and many researchers think of gastrointestinal healing in terms of these phases (van der Vijver et al., 2012). The early stages of inflammation are characterized by innate immune cell activation, such as neutrophils and macrophages (Marks et al., 2017). Neutrophils increase hypoxia in inflammatory environments as they consume more oxygen than other cells for the antimicrobial oxidative burst. These effects potentially lead to microbiota shifts. A number of anti-inflammatory interventions and their role in preventing AL have been investigated in animals and humans, which gave inconsistent results (Foppa et al., 2020b). Inflammation-related gut microbiota changes might be a mediator for the deleterious effects of AL on anastomotic healing.

#### Surgical stress

So far, there is no evidence proving the relationship between surgical stress and AL. Patients who undergo surgery should experience a complicated endocrine and metabolic shift to surgical stress. Stress and the gut microbiota are linked through the bidirectional microbiota–gut–brain axis. Stress can affect the microbiota composition, and the microbiota can influence the host’s response to stress (Malan-Muller et al., 2018). Stress is not only able to shift the microbiota but also increases the intestinal permeability in favor of microorganism translocation (Rodiño-Janeiro et al., 2015).

Using quorum sensing, opportunistic pathogens can sense host environmental changes and respond by inducing a phenotypic shift in their virulence (Seal et al., 2010). The release of host stress factors activates bacterial virulence genes and transforms the pathogen from an innocuous colonizer into a virulent and invasive phenotype. These effects play a key and causative role in anastomotic disruption (Luong et al., 2014). Certain bacteria have been identified as able to recognize and respond to host-derived elements during physiological stress, such as *E. coli* and *P. aeruginosa*, all of them being collagenase bacteria (Sperandio et al., 2003; Wu et al., 2005), as well as *Salmonella typhi*, *Yersinia enterocolitica*, and *Campylobacter jejuni* (Lyte, 2014).

#### Exposure to oxygen during surgery

Many species in the gut are facultative or obligate anaerobes. Exposure to oxygen during bowel sections or anastomosis construction could significantly deplete these species. The disappearance of the beneficial obligate anaerobes (*Bacteroides*) and the occurrence of detrimental facultative anaerobes (*Enterococcus*) have been observed after opening the bowel in a rat model (Shogan B et al., 2014). It is suspected that laparoscopic surgery delivers limited oxygen to the intestinal lumen, which might have a weak effect on this issue compared to open surgery. However, there is no study available on this subject. One study found that laparoscopic surgery appears to be associated with better intra- and postoperative intestinal tissue oxygen pressure. On the other hand, high-pressure pneumoperitoneum may impair the postoperative intestinal tissue oxygen pressure, which might also affect obligate anaerobes in the gut. We look forward to these types of studies in order to discover more information on gut microbiota and laparoscopic surgery in the future.

## Colonization of pathogens on the gut mucosa

**Figure 2:**
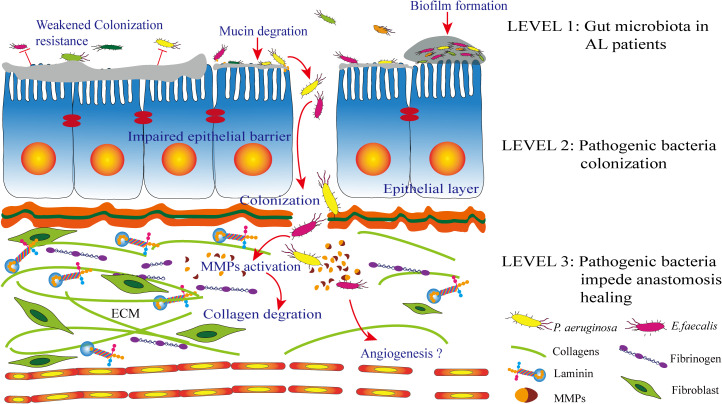
*Level 1*: patients with anastomotic leak (AL) are characterized by an altered gut microbiota. *Level 2*: the wound healing tissue is a desirable environment for collagenolytic strains. The extracellular matrix (ECM) on the anastomosis site, which includes collagen, fibrinogen, and laminin. Bacterial adhesin proteins are essential for the adhesion process. In addition, pathogen biofilm formation and a weakened colonization resistance from commensal bacteria together contribute to this process together. *Level 3*: following successful colonization, the pathogen can degrade mucin and inhibit epithelial cell repair. Most importantly, pathogens such as *Enterococcus faecalis* and *Pseudomonas aeruginosa* have been identified to express proteases and then activate matrix metalloproteinases (MMPs), which contribute to submucosal ECM breakdown and lead to AL.

Surgical resection of the intestinal tube and anastomotic repair lead to alterations in the gut microbiota, which are mainly related to intestinal tissues, but not the microbiota in luminal contents. This phenomenon suggests that tissue-specific microbial taxa possess an adhesion ability, which may partly illustrate their tendency toward wound tissues (Wang et al., 2018). The “adhesin” proteins of bacteria are essential for their adhesion effect on the host cell or the extracellular matrix (ECM) (Singh et al., 2012a). On the other hand, the high colonization rate of collagenolytic strains on healing anastomotic wounds after surgery suggests that the healing anastomotic environment is a favorable niche for these strains. *E. faecalis* is a low-abundance commensal organism comprising less than 1% of the adult gut microflora. It is unknown why its population significantly (up to 500-fold) increases at the site of colon surgery. The ECM is not present when tissues are intact but exposed to the surgical site. The central fibrous proteins forming parts of the ECM are collagens, fibronectins, and laminins, making these molecules a preferred target for bacterial adhesion (Singh et al., 2012b). In addition, bacteria can quickly and effectively attach to host cells or protect themselves by forming a biofilm to escape the clearance effect (Krachler and Orth, 2013) (**Figure 2**).

### ECM is a prerequisite for bacterial colonization

A healing anastomotic environment is exposed to an ECM that is usually not present when the intestinal wall is intact. These ECM components include collagen, fibrinogen, and laminin (Flores-Mireles et al., 2014), in which bacterial adhesion is enhanced in the presence of serum (Nallapareddy and Murray, 2008), another typical exposure cue at the anastomotic site of surgical injury.

Some bacteria colonize intestinal tissues depending on their ability to feed off mucus, a source of enriched organic phosphate on the surface of the mucosa (Tailford et al., 2015). Mucus depletion leads to collagen exposure on anastomotic sites, allowing the pathogen to colonize and express collagenases (Wiegerinck et al., 2018). A classic study found that irradiated rats were more inclined to develop AL when treated with *P. aeruginosa*. This is due to the non-irradiated rats maintaining an intact mucus layer to defend against the colonization of pathogens and the virulence effects of the collagenolytic activity phenotype (Hyoju et al., 2018).

#### Collagen

Collagen is the primary ECM component that plays a critical role in wound healing. Fibroblasts in the connective tissue secrete collagens. However, epithelial cells also produce certain types of collagens during the wound healing process (Ricard-Blum, 2011). Collagen-binding adhesins were found to exist in some pathogenic bacteria. These proteins are essential for bacteria to adhere to collagen. Most adhesin–host protein interactions were found in Gram-negative bacteria with collagen types I, IV, and V, such as the saccharides of *P. aeruginosa* (Singh et al., 2012a).

#### Fibronectin

Fibronectin is a glycoprotein on cell surfaces that can also be found in body fluids. The primary function of fibronectin is to connect the cell and the ECM, then recognize the tissue structure. Over 40 years ago, Staphylococcus aureus *was discovered to have the ability to* bind to fibronectin. This first study reported bacterial binding capacity to the ECM (Kuusela, 1978). Fibronectin-binding proteins on bacterial cells are significant for the adhesion ability of bacteria. Inactivation of the respective fibronectin-binding protein genes leads to a diminished or abolished adhesion effect (Schwarz-Linek et al., 2003).

#### Laminin

Laminin is a multifunctional molecule with numerous heterotrimeric isoforms that are differentially distributed in different types of tissues. Laminin is in charge of keeping the structural scaffold of the tissue, mediating cell migration, and signaling transduction (Singh et al., 2012a). The existence of laminin-binding proteins has been identified in Gram-negative pathogens such as E. coli, Neisseria meningitides, Haemophilus influenza, Y. enterocolitica, Helicobacter pylori, and Borrelia burgdorferi (Singh et al., 2012a).

### AL-related pathogens equipped with adhesins

A series of adhesin proteins have been identified as present on the surface of pathogens. These molecules mediate the adherence ability of bacteria to colonize the wound healing tissue. In addition, adhesins help pathogens form a biofilm in order to deal with host clearance strategies (Berne et al., 2015).

#### Enterococcus faecalis

*E. faecalis* is a commensal bacterium that mainly colonizes the gastrointestinal tract. In patients with compromised immunity, *E. faecalis* transforms into an opportunistic pathogen and then causes a series of infectious diseases, such as wound infection, hospital-acquired infections, and urinary tract infections. In healthy individuals, *E. faecalis* exists in a low abundance of less than 1% in the adult gut microflora. However, its population can increase 500-fold at the anastomosis site in rats after colon surgery. In addition, this strain isolated from the tissue around the anastomosis site expresses a higher level of collagenase (Christley et al., 2020), performs collagen-degrading and MMP-9-activating activities in intestinal tissue, and contributes to the pathogenesis of anastomotic leak (Shogan BD et al., 2015). Moreover, clinically isolated *E. faecalis* strains showed an adherent capacity to ECM components, such as collagens I, II, IV, and V, fibronectin, vitronectin, and laminin (Tomita and Ike, 2004; Singh et al., 2010). Knockout or antibody blocking experiments confirmed these results, suggesting that successful colonization and infection of tissues by *E. faecalis* depend on an efficient adhesin-mediated adherence to the ECM, particularly collagen (Singh et al., 2010). Clinical evidence shows that patients with endocarditis caused by *E. faecalis* infection can be identified with a high level of collagen adhesins and specific antibodies in serum (Nallapareddy et al., 2000).

#### Pseudomonas aeruginosa

*P. aeruginosa* can cause widespread human diseases and is a leading pathogen of nosocomial infections such as pneumonia, urinary tract infections, and bacteremia, especially in immunocompromised patients (Skariyachan et al., 2018). Clinically isolated strains often present as multi-drug resistant. An early study found that *P. aeruginosa* colonizes the mucus of the respiratory tract of patients with chronic lung disease. A specific adhesin–receptor system realizes this effect. A group of adhesins helps *P.aeruginosa* to attach to epithelial cells or mucins, such as mucoid exopolysaccharide and LPS (Ramphal et al., 1987). *P. aeruginosa* also produces two types of lectins to perform their virulence effects: PA-IL and PA-IIL. These two molecules bind to galactose- and fucose/mannose-containing glycoconjugates (Imberty et al., 2004). In addition, *P. aeruginosa* has been found to adhere to collagens I, II, and IV in the basal lamina (Tsang et al., 2003). A study also demonstrated that the adherence of *P. aeruginosa* to stable ECM or epithelial cells may be less significant than that hidden in the self-build biofilm by binding to mucin, which is characterized by high affinity (Paulsson and Riesbeck, 2018).

### Biofilm formation supports pathogen colonization

Bacterial biofilms are diverse populations of bacteria mixed with a matrix attached to biotic or abiotic surfaces. Biofilms act as a community where the microorganisms cooperate closely as a strategy to defend against clearance resistance (Costerton et al., 1995). Biofilm formation occurs when bacteria accumulate on a biological surface and are enclosed by a polymeric matrix (Costerton et al., 1999). A previous study has emphasized that the colon microbiota protects itself in a biofilm (Wu et al., 2013). Approximately 80% of infectious diseases in the human body are mediated by biofilms (Sender et al., 2016). Biofilms have been demonstrated to contribute to several conditions affecting the gut, including gut wounds (Tytgat et al., 2019).

Bacterial biofilms can enhance bacterium-induced loss of intestinal barrier function (Soler et al., 1999). Since biofilm-mediated conditions allow bacteria to expand on the surface of the intestinal epithelial barrier, a necessary precondition for bacterial invasion triggers subsequent inflammatory responses (Johansson et al., 2014). A previous study found that *P. aeruginosa* can adhere to the respiratory mucosa through its type IV pili and flagellum and then secrete ECM to form a biofilm and secrete toxins damaging to the host cells of the lung epithelium (Maurice et al., 2018). However, the significance of bacterial biofilms in AL has not been determined and needs further investigation.

### Weakened commensal bacterium-induced colonization resistance

Due to preoperative interventions or surgical stress, a perturbation of the gut microbiota is always characterized by low microbial diversity, leading to abnormal metabolic balance and weak colonization resistance to pathogens, which could contribute to the development of AL (Shogan et al., 2013). Generally, the protective effect of the mucous layer against mucosal infections is provided by the commensal bacteria that occupy the microbial niche, where it is difficult for opportunistic pathobionts and enteric pathogens to inflict infection (Li et al., 2015). Therefore, bacterial survival or extinction in the host may be determined by its adaptation to life and the microbial community structure in this layer. For example, the mucous layer is a dynamic structure that undergoes rapid renewal. Microbes have to compete with one another for resources to survive (Bachmann et al., 2017).

A low microbiota diversity can be seen in diabetic and overweight patients, which may partly explain why these patients have a relatively high risk of AL development (Buffie and Pamer, 2013; Shogan BD et al., 2015). Loss of the colonization resistance of the normal microbiota that protects intestinal tissues from invasion by collagenolytic microbes is a prerequisite for strains such as *E. faecalis* in order to predominate at sites of anastomotic tissues (Bachmann et al., 2017).

## The mechanism of pathogens affecting anastomotic healing

Due to the complicated biological processes, it is not easy to build a model of anastomotic healing *in vitro*. In addition, a systematic review claimed that animal studies on AL are limited (Yauw et al., 2015). It has been demonstrated that intestinal anastomotic healing is anatomically vague for supervision, compelling surgeons to evaluate the success of anastomosis based merely on the patient’s general wellbeing (Thornton and Barbul, 1997). Not only is this a puzzle in daily clinical practice, but it is also an explanation that knowledge of intestinal healing is very much limited compared to skin wound healing.

Similar to skin wound healing, the anastomotic healing process is also considered to involve four classic stages. The first inflammatory stage is characterized by the activation of neutrophils, macrophages, fibroblasts, and platelets and the release of a group of growth factors, as well as protease activation to increase the collagenolytic profile. This phase would be affected by the gut microbiota and surgical manipulation. Indeed, 2 days following surgery, colorectal anastomosis is only at 30% of its initial strength (Guyton et al., 2016). The next proliferative stage is collagen deposition from fibroblasts, smooth muscle cells, and epithelial cells. In the final remodeling stage, the microscopic structure of the anastomosis is remodeled by collagenase and other protein enzymes to increase its elasticity and contractile capacity (Chang et al., 2015). It has been suggested that the gut microbiota significantly positively or negatively affects gut wound healing in the inflammatory and proliferative stages (Meneghin and Hogaboam, 2007).

The illustration above explains why researchers draw direct parallels between anastomotic and skin wound healing. However, are these two processes identical, or should they be treated as two separate entities? Nevertheless, due to apparent differences between the skin and bowel healing processes, caution should be taken when studying anastomotic healing. One aspect is heavy gastrointestinal colonization with bacteria, which could lead to a higher possibility of infectious complications. In addition, the load and the composition of the skin and gut microbiota flora are entirely different, which can play distinct roles in wound healing (Grice and Segre, 2011). Finally, the collagen subtypes in the gastrointestinal tract, mainly collagens I, III, and V, are secreted by smooth muscle cells and fibroblasts, while collagens I and III are secreted by fibroblasts only in the skin (Bosmans et al., 2015).

Moreover, it should be noted that the relative importance of the four bowel wall layers, i.e., mucosa, submucosa, muscularis propria, and serosa, has not been determined in anastomotic healing. Nevertheless, the mucosa and submucosa are closer to the gut microbiota. Here, we illustrate the already known anastomotic healing mechanism and the potential contribution of the gut microbiota to this process based on the anatomic hierarchy of the intestinal wall (**Figure 2**).

### Mucin layer

The colon epithelia are protected by a two-level mucous layer formed by the mixture of the MUC2 mucin and a limited number of other components secreted from goblet cells (Hand, 2016). The inner colonic mucous layer is about 200 μm in humans, which is impenetrable to bacteria, and this layer is quickly renewed (Hansson, 2012). The inner layer is covered by a non-attached outer mucous layer that is penetrable by colonized bacteria, which use this layer as their habitat. A clinical study found a higher abundance of the mucin-degrading taxa of Lachnospiraceae and Bacteroidaceae in the anastomotic tissue of patients with AL. The abundance of these bacteria could be used to predict AL (van Praagh et al., 2019).

A functional study found that *Muc2* gene knockout mice lack a mucous layer; therefore, the colonized bacteria make contact with the epithelial cells directly in the intestine (Bosmans et al., 2017). In addition, without a mucous layer, *Muc2* knockout mice experience more inflammation, less collagen deposition, and angiogenesis. Thus, the mucous layer promotes the healing of colonic anastomoses. Furthermore, there is a higher bacterial translocation to the mesenteric lymph nodes and spleen in *Muc2* gene knockout mice (Bosmans et al., 2017). It has been proven that colonic ischemia results in mucus detachment, which facilitates a direct connection between bacteria and the epithelium (Grootjans et al., 2013). Certain bacteria have been identified to affect the mucous layer, including *Akkermansia muciniphila* and *Listeria monocytogenes* (Coconnier et al., 1998; Everard et al., 2013). *Bacteroides* and *Blautia*, belonging to the Lachnospiraceae family, are known as mucin degraders (Ouwerkerk et al., 2013). The correlation identified between AL and Lachnospiraceae (van Praagh et al., 2016), a large group of the Lachnospiraceae sequence at the species level, was found to comprise mucin-degrading taxa (*Ruminococcus obeum*, *Ruminococcus gnavus*, and *Ruminococcus torques*) (Sun and Shen, 2018). Other factors affecting the mucous layer are luminal factors such as prostaglandins and SCFAs (Barcelo et al., 2000). SCFAs can modulate the expression profile of epithelial cells, enhancing the production of proteins involved in the biosynthesis of mucin (Finnie et al., 1995). Specifically, butyrate enhances the expression of MUC2, activating the MUC2 promoter and enhancing histone acetylation through histone deacetylase (HDAC) inhibition in cell cultures (Finnie et al., 1995; Burger-van Paassen et al., 2009).

### Epithelial layer

During surgical anastomosis, the distance between two sutures leads to the proximal and distal ends not being meticulously connected circumferentially. However, circumferential linking of the two ends is a prerequisite for primary intestinal wound healing because the first step is epithelialization (Mammen and Matthews, 2003).

A recent study has demonstrated that gut resident bacteria could promote epithelial restitution *via* inducing reactive oxygen species (ROS) generation in epithelial cells (Swanson et al., 2011). Microbiota epithelial cell interactions can activate β-catenin signaling, a critical factor in regulating epithelial cell proliferation (Sun et al., 2004). *A. muciniphila* plays a significant role in wound healing by stimulating signaling pathways to increase the migration and proliferation of epithelial cells. Mechanically, molecular FPR1 and neutrophilic NADPH oxidase (NOX2) is needed to deplete local oxygen that leads to the enrichment of anaerobic bacteria, which is beneficial to wound healing (Alam et al., 2016).

Bacteria ferment fibers to produce SCFAs, including acetic acid, propionic acid, butyric acid, valeric acid, and isovaleric acid, which comprise the primary fuel for colonocytes and exert an immediate nutritive effect on the colonic mucosa (Parada Venegas et al., 2019). Butyrate is the primary energy resource for colonocytes to carry out re-epithelialization and maintain viability and barrier integrity (Canani et al., 2011). In addition, butyrate can downregulate pro-inflammatory cytokines (Arvans et al., 2005). However, a clinical study found that the concentrations of gut SCFAs decreased after surgery (Ohigashi et al., 2013b). This effect might be attributed to the disturbed microbial stability, which may affect the normal metabolic balance in the gut, i.e., a decrease in butyrate production and energy deprivation. Butyrate is the preferred fuel utilized by colon epithelial cells and has been shown to promote the proliferation of epithelial cells and enhance the intestinal barrier by increasing the expression of tight junction proteins, such as claudin-1 and zonula occludens-1. In addition, these organic acids are usually the most abundant in the intestinal tract, which helps maintain the acidity of the tract to inhibit the growth of pathogenic bacteria. Epithelial cells express receptors for SCFAs, such as G protein-coupled receptor 41 (GPR41), GPR43, and GPR109a. The activation of GPR41/GPR43 by SCFAs upregulates the production of cytokines and chemokines by the colonic epithelium, contributing to the clearance of pathogenic bacteria (Kim et al., 2013). In a word, the decreased abundance of obligate anaerobes may have caused the reduced concentrations of SCFAs, which in turn may have increased the number of facultative and aerobic bacteria (Ohigashi et al., 2013b).

Animal studies have provided proof that an intraluminal supplement of SCFAs results in stronger colonic anastomoses (Rolandelli et al., 1986). The application of butyrate-producing bacteria assists in epithelial repair (Scales and Huffnagle, 2013). Until now, no clinical investigation has been performed to evaluate the positive effect of SCFAs on AL.

### Submucosal layer

Whenever a colorectal resection is performed, all four layers are transected and then an anastomosis can be created. Researchers now recognize that the submucosal layer is the main structure in anastomotic healing (Bosmans et al., 2015). This layer of the bowel is the most tensile-resistant fibrous layer consisting mainly of elastin fibers and collagen, the most tensile layers (Thompson et al., 2006). This layer is the primary resource of fibroblasts that changes into an active state after gastrointestinal surgery to produce and deposit collagen.

It is widely accepted that submucosal collagen degradation occurs immediately after surgery, followed by *de novo* collagen synthesis. This collagenous equivalence is crucial in routine wound healing (Chowcat et al., 1988). As early as the 1990s, this balance was believed to support ECM remodeling and enhance tissue strength, leading to high-quality anastomotic healing (Martens and Hendriks, 1991). Nowadays, researchers agree that, in all wounds, protease activity is an indication of wound healing as a balance between collagen synthesis and degradation (McCarty and Percival, 2013). Early in the 1980s, the theory that the increased collagenase production of bacteria contributes to AL emerged. When applying a collagenase inhibitor, an improvement in the breaking strength and anastomotic burst pressure was achieved in a rat model (Young and Wheeler, 1983). Clinical evidence from a randomized controlled trial (RCT) demonstrated that the application of a collagenase inhibitor significantly decreased the radiological and clinical AL rates (Young and Wheeler, 1984).

Several pathogens have been identified to express collagenase, including *E. faecalis*, *P. aeruginosa*, and *Serratia marcescens*, all of which have been shown to contribute to the development of AL (Olivas et al., 2012; Shogan BD et al., 2015). A cause and effect investigation found that the gelatinase (GelE) of *E. faecalis* is a protease with a broad substrate. GelE is responsible for the collagenolytic activity of *E. faecalis*, which can degrade collagen and fibrin (Boiko et al., 2009). Because *E. faecalis* has to use fibrinogen to support its growth, it requires collagenolytic activity to cleave the nascent molecule (Flores-Mireles et al., 2014). Here, we illustrate two types of proteases that have been reported to be involved in AL.

#### Matrix metalloproteinases

MMPs are essential in ECM reorganization during the remodeling stage. However, the overactivation of MMPs might lead to AL (Krarup et al., 2013). Patients with poor anastomotic healing display a lower collagen type I/III ratio than others. In addition, significantly higher levels of MMP-2 and MMP-9 in the submucosal layer were found even in the distant sector bowel wall in patients with AL (Stumpf et al., 2005). There is also clinical evidence of MMP-8 and MMP-9 being significantly higher in postoperative peritoneal fluid in patients who developed AL (Pasternak et al., 2010).

An early study identified that some bacterial proteinases could perform MMP-activating function, which may play a significant role in wound healing by remodeling the ECM during bacterial infections (Okamoto et al., 1997). For example, *E. faecalis* can activate MMP-9 through its collagenolytic ability to aggravate the tissue breakdown effect (Shogan BD et al., 2015).

#### Plasminogen

Bacterial-mediated plasminogen (PLG) activation also plays an essential role in the pathogenesis of AL (Jacobson et al., 2021). Interestingly, *P. aeruginosa* and *E. faecalis* have developed the ability to get the host PLG system up to a high level, leading to collagen lysis (Lahteenmaki et al., 2005). In addition, a broad group of pathogens expresses plasmin receptors to immobilize plasmin on the bacterial cell surface. By this means, pathogens enhance PLG activation with the help of mammalian PLG activators. Tranexamic acid (TXA) is an antifibrinolytic lysine analog that inhibits PLG from binding to bacterial cellular receptors. The pharmacological application of TXA successfully prevented AL by targeting the pathogen-induced PLG activation (Jacobson et al., 2021).

### Potential effects of the gut microbiota on angiogenesis on the anastomosis site

Angiogenesis is a complicated process involving endothelial and mesenchymal cell types (Rieder and Fiocchi, 2009). Microbiota-induced angiogenesis is a critical step for proper anastomosis wound healing by forming new blood vessels. In 2002, a study demonstrated that the microbiota helps construct a microvascular network in the submucosa. The authors showed that Bacteroides induce angiogenesis in the small intestine via Paneth cells (Stappenbeck et al., 2002). The gut microbiota can selectively stimulate mucosal endothelial and submucosal mesenchymal cells to induce specific angiogenic activation (Schirbel et al., 2013). Another striking experiment in rodents has shown that gut microbes are crucial to regulating the vascularization of the intestinal mucosa and that they affect wound healing processes (Reinhardt et al., 2012). Commensal bacteria in the gut regulate angiogenesis to restrain intestinal inflammation and promote mucosal tissue healing by vascular endothelial growth factor (VEGFR) signaling in the inflammation stage of wound healing (Chen et al., 2013). In addition, gut microbes alleviated radiation-induced intestinal injury and improved the survival rates in a murine irradiation model. This effect is realized by upregulating the expression of VEGF in the small intestine tissue of irradiated mice (Cui et al., 2017). These studies support the theory that gut microbiota might mediate angiogenesis.

## Future perspectives and therapeutic approaches

Until now, the pathophysiology of anastomotic healing is still not fully understood. This could be attributed to the complex biological processes of anastomotic healing, which cannot be mimicked appropriately either *in vitro* or in animal research. However, the discovered molecular mechanism of anastomotic healing is less pervasive than thought.

This dilemma is perhaps due to the lack of techniques to create anastomosis and to observe the healing progress directly in an internal organ, which is close to a clinically relevant manner. In addition, clinical evidence on the gut microbiota and AL is still insufficient. This is due to the technique or ethical restrictions, which make it impossible to obtain anastomosis site tissue samples postoperatively. Thus, investigation on the pathogenesis of AL would progress markedly by performing further analyses using human anastomotic tissues during and after surgery (Shogan et al., 2014). Furthermore, with technological progress, gut organoids can be used instead of an animal model to determine the molecular process of anastomotic healing (Sato and Clevers, 2013).

Gut microbiota-induced AL is a continuous process with three aforementioned levels. However, we have not identified the definite driver of the altered gut microbiota during anastomotic healing. In addition, the exact molecular mechanism of the bacterium-induced biological effects on epithelial or mesenchymal cells on the anastomotic tissue that affects healing is not well understood. Thus, strategies targeting the disruption of bacteria–host interaction with anti-adhesion therapy should be effective therapeutic methods. These strategies include receptor blocking, inhibiting surface receptor biogenesis on the pathogen receptor or host cell, and inhibiting biofilm formation.

Several studies have demonstrated that the low concentrations of certain antibiotics under the antibacterial effect can lead to various physicochemical properties on the bacterial cell surface and inhibit bacterial adhesion from host cells. This effect of antibiotics is suspected through altered protein production of partial or incorrect protein folding, then impairing the assembly of bacterial adhesins (Wojnicz and Jankowski, 2007). Inhibition of the host receptor biogenesis has also been proven effective in preventing bacteria from adhering to the host. Many bacterial adhesins depend on host membrane glycosphingolipids (GSLs) to perform their function. Depletion of the host GSLs has been proposed as an efficient strategy for preventing infections (Hartlova et al., 2010). The human body’s autogenous defense strategy against bacteria is dependent on sugars, which act as decoys for bacterial cell surface receptors. Receptor analogs can be used as competition-based strategies, such as sugar-related glycomimetics and inhibitors (Shoaf-Sweeney and Hutkins, 2009).

In addition, polyphosphate (PPi-6) also markedly attenuated biofilm production and prevented anastomotic abscess formation and leakage in mice. This effect decreased the *S. marcescens* and *P. aeruginosa* colonization and collagenase activity in anastomotic tissues after exposure to these pathogens, but did not affect normal growth and did not lead to bacterial resistance (Hyoju et al., 2018). *E. faecalis* has been a specific AL pathogen for many years. It is particularly significant as the most common pathogen that can be cultured from a leaking anastomotic tissue from patients (Belmouhand et al., 2018). Unfortunately, it cannot be eradicated by antibiotics (Goh et al., 2017). Such anti-adhesion could be a promising strategy for preventing AL because it does not bring about bacterial resistance, which is a difficult challenge for conventional antimicrobial methods.

Clinical evidence on the gut microbiota and AL is still lacking. Targeting bacteria–host interaction using anti-adhesion therapy should be an effective therapeutic method. Further investigation focusing on the gut microbiota should be a promising avenue for uncovering the elusive cause of AL.

## Author contributions

YL contributed to content design, data collection, and manuscript writing. BL is in charge of data collection and drawing the figures. YW was responsible for reviewing the manuscript and making suggestions. All authors contributed to the article and approved the submitted version.

## Funding

This study was supported by the Ningbo Clinical Research Center for Digestive System Tumors (2019A21003) and the National Natural Science Foundation of China (NSFC81970466).

## Conflict of interest

The authors declare that the research was conducted in the absence of any commercial or financial relationships that could be construed as a potential conflict of interest.

## Publisher’s note

All claims expressed in this article are solely those of the authors and do not necessarily represent those of their affiliated organizations, or those of the publisher, the editors and the reviewers. Any product that may be evaluated in this article, or claim that may be made by its manufacturer, is not guaranteed or endorsed by the publisher.
